# Nutrition affects insect susceptibility to *Bt* toxins

**DOI:** 10.1038/srep39705

**Published:** 2017-01-03

**Authors:** Carrie A. Deans, Spencer T. Behmer, Ashley E. Tessnow, Patricia Tamez-Guerra, Marianne Pusztai-Carey, Gregory A. Sword

**Affiliations:** 1 Department of Entomology, Texas A&M University, College Station, TX 77843, USA; 2 Department of Entomology, University of Minnesota, St. Paul, MN 55108, USA; 3 Ecology & Evolutionary Biology Graduate Program, Texas A&M University, College Station, TX 77843, USA; 4 LIV-DEMI, Facultad de Ciencias Biológicas, Universidad Autónoma de Nuevo León, San Nicolás de los Garza, N.L. 66455, México; 5 Department of Biochemistry, Case Western Reserve University, Cleveland, OH 44106, USA

## Abstract

Pesticide resistance represents a major challenge to global food production. The spread of resistance alleles is the primary explanation for observations of reduced pesticide efficacy over time, but the potential for gene-by-environment interactions (plasticity) to mediate susceptibility has largely been overlooked. Here we show that nutrition is an environmental factor that affects susceptibility to *Bt* toxins. Protein and carbohydrates are two key macronutrients for insect herbivores, and the polyphagous pest *Helicoverpa zea* self-selects and performs best on diets that are protein-biased relative to carbohydrates. Despite this, most *Bt* bioassays employ carbohydrate-biased rearing diets. This study explored the effect of diet protein-carbohydrate content on *H. zea* susceptibility to Cry1Ac, a common *Bt* endotoxin. We detected a 100-fold increase in LC_50_ for larvae on optimal versus carbohydrate-biased diets, and significant diet-mediated variation in survival and performance when challenged with Cry1Ac. Our results suggest that *Bt* resistance bioassays that use ecologically- and physiologically-mismatched diets over-estimate susceptibility and under-estimate resistance.

Acreage of transgenic *Bt* crops has increased over 60-fold since their introduction in 1996, with over 1 billion acres planted throughout the world^1^. Due to concerns about the evolution of insect resistance to this technology, the U.S. EPA mandates resistance monitoring for all *Bt* plant-incorporated protectants. Nevertheless, incidents of field-evolved resistance to *Bt* crops have been reported in 5 of the 13 insect pest species examined as of 2013^1^. The overriding assumption in *Bt* resistance monitoring is that genetic factors are primarily responsible for the presence of resistant phenotypes^2^,^3^ (Fig. 1a), as evidenced by the fact that all definitions for the term *resistance* describe a change in susceptibility that is genetically-determined and heritable^4^. This is true even though the underlying genetics responsible for resistance are difficult to identify and rarely known in instances of field-evolved resistance, except for a few species/populations^5^,^6^,^7^,^8^,^9^,^10^,^11^,^12^,^13^. We should note, that our use of the term susceptibility throughout the paper refers only to the extent or likelihood of an organism being negatively affected by a *Bt* trait, and this usage makes no assumptions about the mode of susceptibility or its underlying heritability.

An alternative to this gene-centric view is to acknowledge that resistant phenotypes are not solely dependent on an individual’s genotype, but ultimately on gene expression. The regulation of all genes is, at least to some extent, dependent on environmental factors. These gene-by-environment interactions allow a single genotype (individual) to produce a range of phenotypes across different environmental conditions; a phenomenon referred to as phenotypic plasticity. As such, an insect could exhibit low susceptibility to *Bt* in one environment and high susceptibility in another (Fig. 1b). Phenotypic plasticity is widespread across both traits and organisms^14^,^15^,^16^,^17^,^18^ and is likely to occur when populations experience a variable environment with contrasting fitness advantages of specific phenotypes across different environments^19^,^20^,^21^,^22^,^23^,^24^,^25^. Nutrition is a pervasive environmental factor that meets both these criteria, as plant nutrient content is often highly variable^26^,^27^,^28^,^29^ and the nutritional quality of plant resources, particularly the concentration and balance of protein and digestible carbohydrates (henceforth carbohydrates), has strong effects on insect performance, including growth rate, reproduction^30^,^31^, and detoxification ability^32^,^33^. Furthermore, because insects actively regulate their intake of both protein (P) and carbohydrates (C), they can achieve an optimal balance (termed an intake target) by feeding selectively on plant tissues that most closely match their current nutritional needs, or mixing among those that are nutritionally complementary^31^,^34^,^35^. It is also the case that the concentration of plant defensive compounds within and between individual plants can be variable^36^,^37^,^38^,^39^,^40^,^41^ and that plant nutrient content can interact with plant defensive compounds to affect insect herbivore performance^33^,^42^. However, our focus for the current manuscript is on plant nutrients. Once this baseline has been established, it is then possible to study the effects of plant secondary compounds, and interactions between plant nutrients and plant secondary compounds, as environmental factors impacting susceptibility to *Bt* toxins.

Currently the effects of nutrition on insecticide susceptibility and resistance is poorly understood, particularly in agricultural systems where environmentally-mediated effects may have significant economic implications^43^,^44^,^45^. *Helicoverpa zea*, known as the cotton bollworm or corn earworm, is a widespread New World lepidopteran pest targeted by *Bt* transgenic plants. A recent re-examination of *H. zea*’s nutritional requirements highlights a critical disparity between their self-selected protein-to-carbohydrate ratio (P:C) and the P:C ratio of diets routinely used in diet-incorporation *Bt* resistance bioassays. The empirically-determined intake target P:C ratio for *H. zea* is slightly protein-biased, at 1.6:1^46^, but the majority of rearing diets, including commercial diets, for *H. zea* are severely carbohydrate-biased, at around 0.4:1^29^.

We conducted two experiments using *H. zea* as a model to test whether variation in diet protein and carbohydrates impacts susceptibility to Cry1Ac endotoxins, one of the major plant-incorporated insecticides widely expressed in *Bt* cotton and corn. First, we performed 7-day dose-response assays to calculate the LC_50_ for neonates fed either a commercially-available rearing diet or the same diet modified to match *H. zea*’s P:C intake target ratio of 1.6:1. We then expanded our study beyond 7 days and reared larvae on artificial diets created to mimic the range of P:C ratios and total macronutrient concentrations (P + C) present in different cotton tissues^47^. We hypothesized that diet protein-carbohydrate content would have strong effects on survival and performance across sub-lethal and lethal concentrations of Cry1Ac, and that the diet most closely matching the self-selected P:C ratio of 1.6:1 would confer the greatest survival and performance for larva when challenged with Cry proteins.

## Results

### Experiment 1: Cry1Ac dose response assays on diets with different protein-carbohydrate content

**Figure 2:**
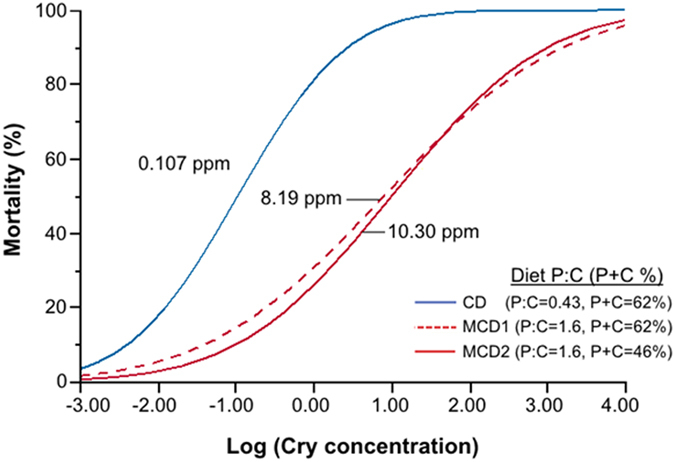
Cry1Ac Dose-Response Assay. Dose-response curves for *H. zea* neonates in diet-incorporated bioassays (Experiment 1). One of the diets was a commercially available (CD) carbohydrate-biased rearing diet commonly used for *Bt* resistance monitoring. The other two diets were modified (MCD1 and MCD2) to reflect protein-carbohydrate profiles that are more physiologically- and ecologically-relevant and to align with *H. zea*’s self-selected P:C intake target. Mortality represents the average for three trials; the LC_50_ concentrations are listed for each diet (N = 22–28 larvae per concentration).

The LC_50_ concentration of Cry1Ac varied by approximately two orders of magnitude across the three diets (Table 1; Fig. 2). It was lowest on the commercial diet (CD; Southland Products, Lake Village, AR), which had a P:C ratio that was very carbohydrate-biased relative to the self-selected P:C observed for *H. zea*^46^; the total macronutrient content (P + C) of the CD was also high (62%) relative to what is typical for most plant vegetative tissues^47^. In contrast, the LC_50_ value was 75 times higher for neonates fed a modified commercial diet (MCD1) that had a P:C matching the self-selected ratio for *H. zea*, but a macronutrient content similar to the CD. Finally, LC_50_ values were 100 times higher (compared to the CD) when caterpillars were fed a modified diet (MCD2) that had a P:C matching the self-selected ratio of *H. zea*, but a total macronutrient content more in line with most plant vegetative tissues (P + C = 46%). Variability in LC_50_ values was evident between the three trials, but the relative effects of diet were consistent. The LC_50_ values for each diet, across trials one, two and three, were as follows: (1) CD – 0.08, 0.92, 0.08, (2) MCD1 – 4.2, 14.4, 8.5, and (3) MCD2 – 32.3, 18.6, 2.2. Despite this variability, the LC_50_ values for the CD were consistently and considerably lower than either of the MCDs across trials. In fact, the LC_50_ values for the MCD1 were between 15.7–106.5 times greater than the LC_50_ values of the CD values, while the LC_50_ values for the MCD2 were between 20.2–408.8 times greater compared to the CD.

### Experiment 2: Interactions between Cry1Ac and food protein-carbohydrate content

We first examined the effects of Cry1Ac concentration on survival independent of diet protein-carbohydrate content (we pooled individuals from all diets together). Specifically, we employed a Kaplan-Meier survival analysis to analyze time until death; all individuals that pupated were right censored. As expected, there were significant Cry1Ac concentrations effects (*X*^*2*^ = 218.70, df = 4, *P* < 0.001). Post hoc tests indicated that time until death was similar on the control and 0.1 ppm treatments (*X*^*2*^ = 0.01, *P* = 0.924), but that it differed significantly between the 0.1 ppm and 0.6 ppm treatments (*X*^*2*^ = 13.60, *P* < 0.001), the 0.6 and 1 ppm treatments (*X*^*2*^ = 18.33, *P* < 0.001), and the 1 ppm and 3 ppm treatments (*X*^*2*^ = 17.34, *P* < 0.001).

**Figure 3:**
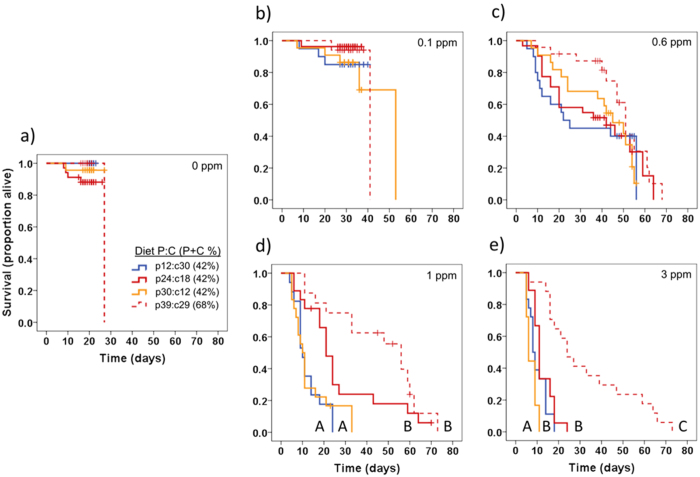
Diet-Cry1Ac Survivorship. Survival plots for larvae reared on diets with different protein-carbohydrate content, at different Cry1Ac concentrations (Experiment 2). Four diets (p12:c30, p24:c18, p30:c12 and p39:c28) and five Cry1Ac concentrations were tested: (**a**) no Cry1Ac. (**b**) 0.1 ppm, (**c**) 0.6 ppm, (**d**) 1 ppm, and (**e**) 3 ppm (N = 16–34 larvae per treatment). Hatch marks indicate pupation events, and line endpoints indicate the point in time where all individuals had either pupated or died. Different letters indicate post hoc differences between the four diets within each Cry1Ac concentration (α = 0.05).

To understand how diet protein-carbohydrate content might mediate these strong Cry1Ac concentration effects, we next compared survival between our four P:C diets within each Cry1Ac concentration. On the diets lacking Cry1Ac (Fig. 3a; *X*^*2*^ = 4.56, *P* = 0.207), and the diets with Cry1Ac at 0.1 ppm (Fig. 3b; *X*^*2*^ = 2.05, *P* = 0.563) and 0.6 ppm (Fig. 3c; *X*^*2*^ = 4.12, *P* = 0.249), there were no significant differences in time until death as a function of diet protein-carbohydrate content. In contrast, significant effects were observed when diets contained Cry1Ac at 1 ppm (*X*^*2*^ = 30.23, *P* < 0.001) and 3 ppm (*X*^*2*^ = 46.69, *P* < 0.001). On the treatments containing 1 ppm Cry1Ac (Fig. 3d), caterpillars lived longest on the p24:c18 and p39:c29 diets; the two diets with a P:C that most closely matched *H. zea*’s self-selected ratio^46^ (1.6:1). On the treatments with 3 ppm Cry1Ac (Fig. 3e), caterpillars lived longest on the p39:c29, the diet with a P:C closest to the *H. zea* intake target but a higher total macronutrient concentration, and shortest on the carbohydrate-biased diet (p12:c30). Larval survival was intermediate on diets that were protein-biased and had total macronutrient content of 42% (p24:c18 and p30:c12).

We also examined pupation success across all Cry1Ac concentrations and diet P:C combinations by scoring larvae as either pupating or not pupating, and then analyzed these data using a logistic regression analysis. We observed a significant Cry1Ac concentration effect (*X*^*2*^ = 292.51, *P* < 0.001), but there was no significant diet P:C effect (*X*^*2*^ = 0.000, *P* = 1.000), or Cry1Ac-by-diet P:C interaction (*X*^*2*^ = 15.10, *P* = 0.236). Odds ratios indicated pupal success was best in the absence of Cry1Ac (Table S1), and that all Cry1Ac treatments differed from one another except for the 1 and 3 ppm treatments (which had equally low pupation).

**Figure 4:**
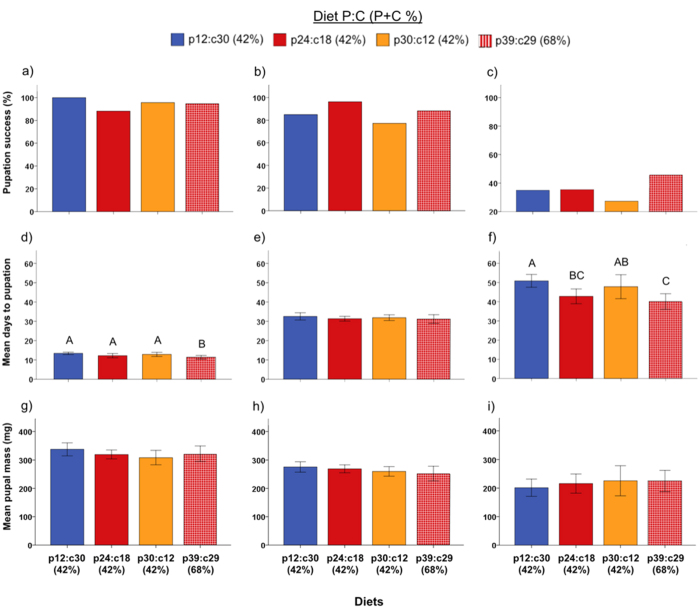
Diet-Cry1Ac Performance. Pupation success (N = 16–34 larvae per treatment), larval development time (N = 6–30 larvae per treatment), and pupal mass (N = 6–32 larvae per treatment) for larvae reared on diets with different protein-carbohydrate content, at different Cry1Ac concentrations (Experiment 2). Development (mean ± SEM) and pupal mass (mean ± SEM) were only recorded for larvae that pupated. Each column represents one of the three sub-lethal Cry1Ac concentrations: no Cry1Ac (panels a, d and g), 0.1 ppm (panels b, e and h) and 0.6 ppm (panels c, f and i). Within each panel the four diets are shown (p12:c30, p24:c18, p30:c12 and p39:c28). Different letters indicate significant differences between treatments (α = 0.05).

Because high mortality was observed on the 1 and 3 ppm treatments, analysis of developmental time from egg to pupation, pupal mass, and consumption was restricted to the controls (no Cry1Ac), 0.1 and 0.6 ppm treatments. Developmental time (egg to pupa) increased as Cry1Ac concentration increased (*X*^*2*^ = 260.56, *P* < 0.001), and each treatment was statistically different from one another (no Cry versus 0.1 ppm: *X*^*2*^ = 158.23, *P* < 0.001; 0.1 ppm versus 0.6 ppm: *X*^*2*^ = 77.17, *P* < 0.001). We thus examined development time on each diet P:C within each Cry1Ac concentration. In the control treatments (no Cry1Ac), a significant difference was observed (*X*^*2*^ = 9.32, *P* = 0.025). Development was fastest on the high macronutrient diet (p39:c29), and statistically similar for the three other diets, which each had a total macronutrient content of 42% (Fig. 4d). There was no difference in developmental time when diets contained 0.1 ppm of Cry1Ac (*X*^*2*^ = 1.93, *P* = 0.588; Fig. 4e), but at 0.6 ppm, significant diet P:C effects were observed (*X*^*2*^ = 13.46, *P* = 0.004). At 0.6 ppm Cry1Ac, development was fastest on the p39:c29 diet and intermediate on the p24:c18 diet (the two diets closest to the intake target P:C ratio), but was equally slow on the carbohydrate-biased p12:c30 and protein-biased p30:c12 diets (Fig. 4f).

There was a significant effect of Cry1Ac concentration on pupal mass (F_2_ = 62.89, *P* < 0.001), but no diet effect (F_3_ = 0.13, *P* = 0.945), or interaction between diet P:C and Cry1Ac concentration (F_6_ = 0.81, *P* = 0.564). A post-hoc comparison between the controls, 0.1 ppm, and 0.6 ppm treatments showed that pupal mass decreased significantly as Cry1Ac concentration increased (Fig. 4d–f).

**Figure 5:**
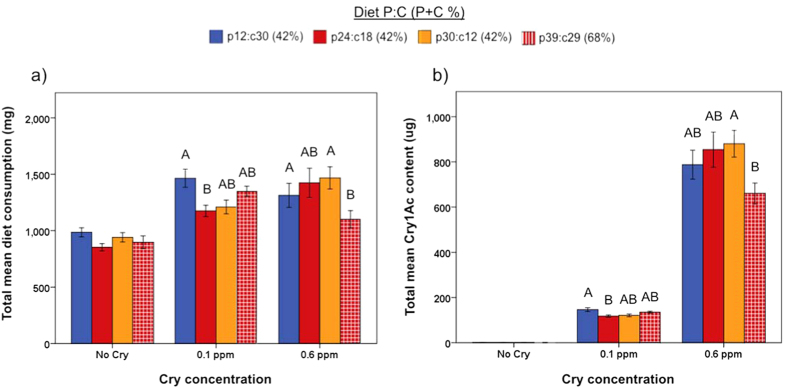
Diet-Cry1Ac Consumption. Total food consumption and total Cry1Ac consumption for larvae reared on diets with different protein-carbohydrate content, at different Cry1Ac concentrations (Experiment 2). Consumption (mean ± SEM) was only recorded for larvae that pupated (N = 7–28 larvae per treatment). Different letters indicate significant differences between treatments (α = 0.05).

Effects of diet P:C and Cry1Ac concentration on consumption were also investigated; for these analyses only consumption from insects that pupated was analyzed. Total diet consumption was significantly affected by Cry1Ac concentration (ANOVA: F_2_ = 59.49, *P* < 0.001); it increased in the presence of Cry1Ac (Fig. 5a). Additionally, a significant interaction between diet P:C and Cry1Ac concentration was observed (ANOVA: F_6_ = 59.49, *P* = 0.014). In the absence of Cry1Ac, total consumption was not affected by diet P:C content. However, when Cry1Ac was present (0.1 and 0.6 ppm), consumption patterns differed depending on the P:C content of the food (Fig. 5a). Cry1Ac intake mirrored food consumption patterns (Fig. 5b), and as with consumption there was a significant interaction between diet P:C content and Cry1Ac concentration (ANOVA: F_3_ = 5.22, *P* = 0.002). Although total diet consumption increased with Cry1Ac concentration, mass-specific diet consumption, which accounts for larval size and duration of feeding period, revealed a significant interaction between P:C and Cry1Ac concentration (ANOVA: F_6_ = 2.18, *P* = 0.048). However, differences in mass specific consumption, as a function of diet P:C, were only observed on treatments with Cry1Ac at 0.1 ppm (Figure S1). On this Cry1Ac treatment, caterpillars eating the p24:c18 diet had the lowest consumption rate, while those eating the p39:c29 diet had the highest consumption; consumption rates on the other two diets were intermediate.

## Discussion

Our study shows that nutrition can mediate the susceptibility of an economically important insect pest to a *Bt* toxin. We found that both diet P:C and total macronutrient concentration had effects on *H. zea* susceptibility to Cry1Ac, but also that these effects were dependent on Cry1Ac concentration. The importance of diet protein-carbohydrate content was most pronounced in our dose-response assay (Experiment 1). Here we showed that simply changing the P:C from carbohydrate-biased (similar to diets typically used in resistance bioassays) to the ratio that *H. zea* actively selects for caused a 100-fold increase in the LC_50_ of Cry1Ac. Our subsequent study, which examined diet P:C and Cry1Ac interactions throughout the entire larval stage (Experiment 2), showed that diet protein-carbohydrate content mediates larval performance in the presence of sub-lethal and lethal Cry1Ac concentrations. When Cry1Ac was absent, diet macronutrients had minimal impacts on survival and larval performance, which is consistent with other nutritional studies investigating generalist caterpillars^48^,^49^,^50^, including *H. zea*^46^. In contrast, when Cry1Ac was present, larvae reared on the diets that most closely matched their self-selected P:C^46^ showed faster development and better survival.

Originally, Waldbauer *et al*.^51^ inferred that *H. zea* self-selected a P:C of 4:1. However, Deans *et al*.^46^ recently revisited P:C regulation in *H. zea*, using a more robust experimental approach, and recorded a self-selected P:C of 1.6:1. This ratio is more in-line with P:C regulation reported for other lepidopterans^31^,^46^. The p24:c18 and p39:c29 diets in the current study were the closest to this self-selected P:C, and correspondingly *H. zea* larvae on these two diets, when challenged with Cry1Ac, showed the best survival and performance. Simpson and Raubeheimer^29^ reported similar results in locusts, where nymphs forced to consume diets with the allelochemical tannic acid had better survival and performance on the diet that most closely matched their self-selected P:C ratio. In the current study, dietary total macronutrient concentration also emerged as an important variable mediating the effects of Cry1Ac, as larvae on both the p24:c18 and p39:c29 diets at 1 ppm performed best and had similar survival curves. However, when Cry1Ac was present at 3 ppm, larvae on the higher macronutrient p39:c29 diet took significantly longer to die than those on the other three diets. This has potential implications in the field, because delayed mortality and an overall delay in development for larvae exposed to Cry1Ac, suggests that larvae feeding on higher macronutrient tissues^47^ (e.g., cotton seed) may produce greater crop damage. This is supported by our consumption data, which shows that larvae consume more total diet when Cry1Ac is present. However, our mass-specific consumption data suggests this is a function of the extended developmental time associated with Cry1Ac ingestion, as opposed to an increase in feeding rate (Figure S1).

While the current study shows that diet protein-carbohydrate content had significant effects on the survival and performance of a susceptible lab strain of *H. zea*, additional research is necessary to determine if this is likely for other *H. zea* strains. Orpet *et al*.^45^ examined the effect of diet P:C on Cry1Ac susceptibility in two strains of *H. zea* – one selected for susceptibility, the other to be genetically-resistant. They found that the resistant strain had better survival in the presence of Cry1Ac, but that diet P:C did not significantly affect survival for either strain. Shikano and Cory^43^ did find differences in the effect of diet P:C on *Bt*-related mortality for a susceptible and resistant strain of *Trichoplusia ni*. In their study, survival of the susceptible strain increased as diet P:C increased in the presence of *B. thuringiensis*, while the resistant strain had higher mortality on the most protein-biased diet. It is difficult to make direct comparisons between our study and Orpet *et al*.^45^, as they based their diet treatments around an optimal P:C of 4:1 (as documented in Waldbauer *et al*.^51^) rather than the updated 1.6:1 ratio^31^,^46^. Additionally, both Orpet *et al*.^45^ and Shikano and Cory^43^ used spore/protoxin formulations of *Bt* rather than activated toxin, which could have produced different physiological responses in the larvae (i.e., triggering an immune response in addition to detoxification pathways). In any case, the results of these studies indicate that genetic background may alter the impact that diet protein-carbohydrate content has on susceptibility to Cry1Ac, highlighting the need for further investigation into the relative roles of genetic and environmental factors on *Bt* resistance using standardized methodologies that are more physiologically- and ecologically-relevant.

In terms of phenotypic plasticity, environmental effects can have a genetic basis and vary among genotypes^16^,^52^,^53^. This means that while it’s difficult to tie plastic phenotypes to specific genes because they have low heritability, the ability of a phenotype to be plastic (vary along environmental gradients) is in itself a trait, that can be heritable. In fact, this kind if phenotypic flexibility may play an important role as an adaptive intermediate stage in evolution^16^,^54^. As such, observations of differential susceptibility to Cry1Ac exhibited by different genotypes in response to variation in nutritional environment are consistent with the notion of environmentally-mediated plasticity in response to Cry1Ac.

The implications of our data are perhaps most important for informing resistance monitoring methodologies^29^. We now know that *H. zea* larvae in the field are capable of regulating their ingestion of plant macronutrients, and likely do so to match a P:C of 1.6:1^46^. It is also apparent that caterpillars can detect Cry endotoxins in plants and often feed selectively to avoid ingestion of these toxins, increasing the likelihood of sub-lethal exposure^55^,^56^,^57^,^58^.For resistance bioassays to accurately estimate susceptibility in the field, they must adequately simulate the nutritional conditions in the field. However, most diet-based resistance bioassays use diets with extremely carbohydrate-biased ratios. This includes commonly used rearing diets as well as commercially-available wheat-germ-based diets^26^. In fact, Deans *et al*.^26^ showed that all of the most recent studies assessing *Bt* resistance in *H. zea* used carbohydrate-biased diets with P:C ratios below 0.52^59^,^60^. Based on our data, carbohydrate-biased diets such as these have likely overestimated susceptibility in *H. zea* by confounding nutritional stress with Cry toxicity, thereby compromising our ability to detect resistance in the field.

Our study highlights two important and often overlooked characteristics of *Bt* susceptibility. First, susceptibility to *Bt* endotoxins can be a plastic phenotype that is not exclusively genetically-determined. Secondly, heterogeneity in plant protein-carbohydrate content, combined with the ability of insects to regulate protein-carbohydrate intake, provides an opportunity for crop pests to mediate the toxic effects of *Bt* endotoxins. These gene-by-environment interactions have significant implications for how we define, monitor, and manage resistance in the field. Genetic mutations are not the only mechanisms that can reduce susceptibility to *Bt* crops, and while these plastic responses do not currently fall under the definition of *resistance*, they can produce resistant phenotypes capable of undermining the efficacy of *Bt* technology, and ultimately costing growers money. This is particularly true for *H. zea,* which is a species that exhibits highly variable regional responses to *Bt* crops, but the same principles may be broadly applicable to other insects targeted by *Bt* crops.

## Methods

### Insects

Caterpillar eggs (*H. zea*) were purchased from Benzon Research (Carlisle, PA). Upon hatching, neonates were individually transferred, using a fine-tipped paint brush, into their experimental containers. For Experiment 1, a colony was started with one batch of eggs from Benzon Research and larvae were collected from different generations for use in each trial. All larvae were housed in a growth chamber (Model I-66VL; Percival Scientific, Perry, IA, USA) set at 28 °C with a 12:12 L:D cycle for the duration of the study. For Experiment 2, larvae were housed in a growth chamber (Model I-66VL; Percival Scientific, Perry, IA, USA) set at 25 °C with a 14:10 L:D cycle for the duration of the study.

### Cry1Ac Solutions

Trypsin-activated HPLC purified Cry1Ac was produced in the Pusztai-Carey Lab at Case Western Reserve University (Cleveland, OH) and stored at −80 °C. Because Cry endotoxins degrade over time at room temperature, each artificial diet was refrigerated and then thoroughly mixed with the appropriate amount of Cry1Ac stock solution just before feeding.

The concentrations of Cry1Ac stock solutions were standardized so that the same amount of solution was needed to achieve the desired Cry1Ac concentration in the diets. This controlled for the amount of water being added to the diets across Cry1Ac treatments. During diet preparation, the total amount of diet needed to feed all replicates within a single treatment was weighed, and the amount of the corresponding Cry1Ac stock solution needed to achieve the overall Cry1Ac concentration in the diet (ug of Cry1Ac per g of diet) was calculated. Stock solutions were then thawed, the appropriate amounts were added to each diet, and the diet thoroughly mixed before being portioned into each rearing container. Water was added to the control diets in the same amounts as the Cry1Ac treatments.

### Experiment 1: Cry1Ac dose response curves on diets with different protein-carbohydrate content

#### Experimental diets

In this experiment we measured the LC_50_ for Cry1Ac across three diets that differed in their protein-carbohydrate content. We tested a common commercial diet (CD; Southland (Lake Village, AR)), similar in nutritional quality to those used in resistance monitoring assays for *H. zea*. This diet had a carbohydrate-biased P:C ratio of 0.43 and a total macronutrient concentration (P + C) of 62%. This protein-carbohydrate profile is similar to other wheat-germ-based rearing diets^36^. We modified the CD to produce two additional diets. The first modified commercial diet (MCD1) had a P:C ratio of 1.6:1, matching the self-selected P:C ratio for *H. zea*^46^, and the total macronutrient content was kept at 62%. The second modified commercial diet (MCD2) also had a P:C ratio of 1.6:1 but a total macronutrient content of 46%, which approximates the total macronutrient content of cotton vegetative tissue^47^. Modifications were achieved through the addition of cellulose, vitamin-free casein, anti-microbials, and agar. Casein provided a protein source, thus its addition increased the percent protein in the diet. Agar and cellulose were used to dilute the total macronutrient concentration. To maintain similar water content the ratio of dry ingredients to water was standardized according to the control diet. Sorbic acid, methylparaben, and chlortetracycline hydrochloride were added to match the concentrations in the control diet.

#### Experimental protocol

Newly hatched neonates were individually placed into wells in a 96 well plate. Each well contained one of the three diets outlined above with one of the following seven concentrations of Cry1Ac incorporated into the diet: 0, 0.01, 0.1, 1.0, 10, 100 and 1000 ppm (ug/g). For the 0 ppm treatment, an equivalent amount of distilled water was added to the diet. We performed three trials and had 10 neonate replicates per Cry1Ac concentration for each diet in each trail.

### Experiment 2: Interactions between Cry1Ac and food protein-carbohydrate content

#### Experimental diets

To maintain relevance to natural conditions, the protein (P) and carbohydrate (C) content, as well as the total macronutrient concentrations (P + C), of our experimental diets in this experiment were selected to match the nutritional value of different cotton plant tissues available to *H. zea* larvae in a typical cotton field^47^. We tested three diet P:C ratios, which contained a total macronutrient concentration (P + C) of 42% (by dry mass). The first diet (p24:c18) contained protein and carbohydrates in a ratio that approximated the published self-regulated P:C intake target for *H. zea*^46^. The second (p12:c30) and third (p30:c12) diets were carbohydrate-biased and protein-biased, respectively, to the first diet. We also tested a fourth diet (p39:c29). The P:C ratio of this diet matched that of the 24:18 diet but contained a higher total macronutrients concentration (P + C = 68%) relative to the other three diets (P + C = 42%).

#### Experimental protocol

Upon hatching, neonates were individually placed, using a fine-tipped paint brush, into 1 oz. clear condiment cups with mesh lids. Each cup contained one of four experimental diets that varied in protein-to-carbohydrate ratio (P:C), and one of four different Cry1Ac concentrations (see below), resulting in a total of twelve different treatments (16–34 larvae per treatment).

Within each of our four diets we tested four different Cry1Ac concentrations: 0.1, 0.6, 1.0 and 3.0 ppm (ug/g), which simulated a range of lethality based on preliminary studies using the same strain of *H. zea*. Cry1Ac stock solutions were mixed with fresh diet at each feeding to achieve the desired concentration of Cry1Ac within the diet, and larvae were given fresh diets a minimum of every four days.

Larvae were fed on their respective diets from hatching, and monitored daily until pupation. Survival was recorded for all treatments, and for larva that pupated we recorded pupation success, larval developmental time, and pupal mass. Consumption was also recorded for all larvae that pupated. Although caterpillars consumed wet agar blocks, consumption was calculated on a dry weight basis (=start dry mass − end dry mass). We recorded the wet mass of each diet block per replicate and then the dry mass of any uneaten diet. Separately, we created a wet-to-dry mass regression by recording the wet mass of diet blocks (not used in the experiment) and their subsequent dry mass after drying in a freeze-dryer. We then used this regression equation to back-calculate the starting dry mass of the diet initially supplied to each replicate. We calculated consumption by subtracting the dry mass of any uneaten diet from the calculated initial dry mass.

### Data Analysis

For Experiment 1, the LC_50_ estimates and dose response curves were calculated using a simple probit analysis in JMP^®^ (SAS Institute). For Experiment 2, a Kaplan-Meier survival analysis (Mantel-Cox test) was used to determine differences in larval survival distributions and developmental time (time to pupation) across treatments. This allowed us to look for the main effects of diet, Cry1Ac concentrations, and interactions between the two. A two-way ANOVA was used to test for differences in pupal mass and consumption. A logistic regression was used to determine the effects of diet and Cry1Ac concentration on pupation success. All these analyses were done using SPSS version 21 for Windows (SPSS Inc., Chicago, IL, USA).

## Additional Information

**How to cite this article**: Deans, C. A. *et al*. Nutrition affects insect susceptibility to *Bt* toxins. *Sci. Rep.* **7**, 39705; doi: 10.1038/srep39705 (2017).

**Publisher's note:** Springer Nature remains neutral with regard to jurisdictional claims in published maps and institutional affiliations.

## Supplementary Material

Supplemental Table S1

## Figures and Tables

**Figure 1:**
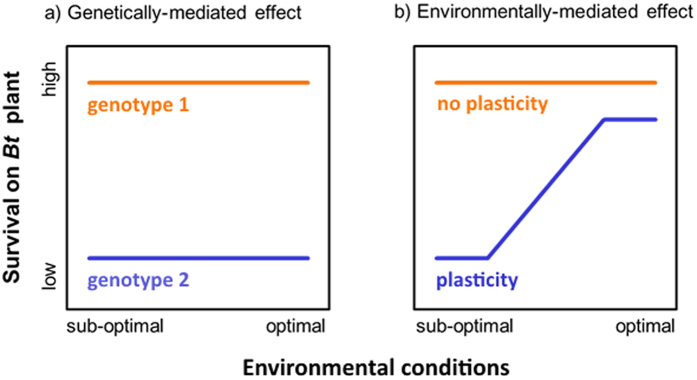
Reaction Norm Model. A graphical phenotypic plasticity model showing how reduced-susceptibility to *Bt* can be mediated by the nutritional environment. Panel (a) shows a genetically-determined effect, while panel (b) shows an environmentally-determined effect.

**Table 1:** The Cry1Ac LC_50_ and 95% confidence intervals (CI) for *H. zea* neonates.

Diet	Diet P:C Ratio	Diet total macronutrient content (P + C)	LC_50_	95% CI
Commercial Diet (CD)	0.43	62%	0.107	0.04–0.32
Modified Commercial Diet #1 (MCD1)	1.6	62%	8.185	1.82–37.15
Modified Commercial Diet #2 (MCD2)	1.6	46%	10.304	2.44–43.65

Three diets were tested. One was a carbohydrate-biased commercial diet (CD) commonly used for rearing caterpillars, and in *Bt*-resistance bioassays. The two other diets were modified versions of the CD. In both cases the modified commercial diets (MCD) had protein-carbohydrate ratios that matched the self-selected intake target of *H. zea* (1.6:1 P:C). The first of these (MCD1) had a total macronutrient (P + C) content equal to the CD. The other (MCD2) had a lower total macronutrient content, which is more in line with total macronutrient content found in plant vegetative tissues.
