# Supplementary material for: Nutrition affects insect susceptibility to Bt toxins
Source: Sci Rep. 2017 Jan 3;7:39705. doi: 10.1038/srep39705 (PMC5206677; doi:10.1038/srep39705)
Supplement: Supplemental Table S1 [file srep39705-s1.doc]

***Title****:* Nutrition affects insect susceptibility to *Bt* toxins.

CARRIE A. DEANS^1,2^, Spencer T. Behmer^1,3^, Ashley E. Tessnow^1^, Patricia Tamez-Guerra^4^, Marianne Pusztai-Carey^5^, and Gregory A. Sword^1,3^

^1^ Department of Entomology, Texas A&M University, College Station, TX 77843, USA

^2^ Department of Entomology, University of Minnesota, St. Paul, MN 55108

^3^ Ecology & Evolutionary Biology Graduate Program, Texas A&M University,

College Station, TX 77843

^4^ LIV-DEMI, Facultad de Ciencias Biológicas, Universidad Autónoma de Nuevo León, San Nicolás de los Garza, N.L. 66455, México.
^5^ Department of Biochemistry, Case Western Reserve University, Cleveland, OH 44106

*Corresponding author: Carrie A. Deans

Entomology Department

Texas A&M University

College Station, TX 77843, USA

Tel: (651) 335-4923

Email: cadeans@tamu.edu

**Table S1.** Odds ratios comparing the effects of Cry1Ac concentrations on pupation success.

| Cry1Ac Treatment   Comparison | Odds Ratio | P Value |
| --- | --- | --- |
| control v. 0.1 ppm | 170.68 | 0.040 |
| control v. 0.6 ppm | 2110.14 | <0.001 |
| control v. 1 ppm | 638624.81 | <0.001 |
| control v. 3 ppm | 6.94e+11 | <0.001 |
| 0.1 v. 0.6 ppm | 1236 | <0.001 |
| 0.1 v. 1 ppm | 3741.72 | <0.001 |
| 0.1 v. 3 ppm | 4.0681e+9 | <0.001 |
| 0.6 v. 1 ppm | 302.65 | <0.001 |
| 0.6 v. 3 ppm | 329044933 | <0.001 |
| 1 v. 3 ppm | 1087228.3 | 0.926 |
